# Predictive validity of the UK clinical aptitude test in the final years of medical school: a prospective cohort study

**DOI:** 10.1186/1472-6920-14-88

**Published:** 2014-04-23

**Authors:** Adrian Husbands, Alistair Mathieson, Jonathan Dowell, Jennifer Cleland, Rhoda MacKenzie

**Affiliations:** 1 School of Medicine, University of Dundee, The Mackenzie Building, Kirsty Semple Way, Dundee DD2 4BF, UK; 2 Division of Medical and Dental Education, University of Aberdeen, Polwarth Building, Foresterhill, Aberdeen AB25 2ZD, UK

**Keywords:** UKCAT, Predictive validity, Psychometric, Assessment, Selection, Admissions, Aptitude

## Abstract

**Background:**

The UK Clinical Aptitude Test (UKCAT) was designed to address issues identified with traditional methods of selection. This study aims to examine the predictive validity of the UKCAT and compare this to traditional selection methods in the senior years of medical school. This was a follow-up study of two cohorts of students from two medical schools who had previously taken part in a study examining the predictive validity of the UKCAT in first year.

**Methods:**

The sample consisted of 4^th^ and 5^th^ Year students who commenced their studies at the University of Aberdeen or University of Dundee medical schools in 2007. Data collected were: demographics (gender and age group), UKCAT scores; Universities and Colleges Admissions Service (UCAS) form scores; admission interview scores; Year 4 and 5 degree examination scores. Pearson’s correlations were used to examine the relationships between admissions variables, examination scores, gender and age group, and to select variables for multiple linear regression analysis to predict examination scores.

**Results:**

Ninety-nine and 89 students at Aberdeen medical school from Years 4 and 5 respectively, and 51 Year 4 students in Dundee, were included in the analysis. Neither UCAS form nor interview scores were statistically significant predictors of examination performance. Conversely, the UKCAT yielded statistically significant validity coefficients between .24 and .36 in four of five assessments investigated. Multiple regression analysis showed the UKCAT made a statistically significant unique contribution to variance in examination performance in the senior years.

**Conclusions:**

Results suggest the UKCAT appears to predict performance better in the later years of medical school compared to earlier years and provides modest supportive evidence for the UKCAT’s role in student selection within these institutions. Further research is needed to assess the predictive validity of the UKCAT against professional and behavioural outcomes as the cohort commences working life.

## Background

In the UK the number of applicants with the required academic qualifications outstrips the number of available medical school places. This necessitates defensible means of differentiating between candidates [1]. Unfortunately, measures of achievement, such as A-levels and Highers, while widely used in selection decisions, have been shown to be associated with socioeconomic class [1]. Other traditionally popular methods of selection into medical school, such as references and personal statements have shown similar bias and have little or no predictive validity [2-9]. Moreover, A-levels and Highers appear to be losing their discriminatory power as most applicants to medical school currently achieve near maximum grades [8]. There is therefore a need for additional fair and psychometrically robust admissions measures.

In an attempt to address these issues, the United Kingdom Clinical Aptitude Test (UKCAT)[10] was introduced in 2006. The UKCAT was designed to help ‘…*select students who will perform well in medical or dental school and who will eventually make good doctors or dentists*’ (p.3) [11]. The test addresses a range of cognitive abilities identified as important to the practice of medicine, namely verbal, quantitative and abstract reasoning and decision analysis. As with other aptitude tests worldwide such as the Medical Colleges Aptitude Test (MCAT) [12] and Graduate Australian Medical School Admissions Test (GAMSAT) [13], determining whether the UKCAT is predictive of important outcomes such as medical school examination performance is important to justify its use as a selection tool. However a distinction should be made between validity coefficients observed from mainly pre-clinical assessments undertaken in the early years and those in the latter, clinical years. This is because achievement in the early years is mostly knowledge-based whereas application of this knowledge and clinical decision-making are more heavily weighted in the later years.

Such a distinction was observed by Callahan et al. [14] in a longitudinal study at one North American institution where the authors reported consistent MCAT validity coefficients using combined Grade Point Average (GPA) as a criterion measure, with assessments which included written and clinical assessments. These authors found validity coefficients in the early pre-clinical years of around .45, with a pattern of decline to .30 in the latter years. A similar difference was observed in a meta-analysis by Donnon et al. [12] who found MCAT validity coefficients of .66 for preclinical assessments and .43 to .48 for the later clinical exams. It is therefore possible that the aptitude required to perform well in pre-clinical years may be very different from that required later in later years. This has important implications for how medical schools interpret the UKCAT’s predictive validity as performance in the later clinical years is more likely to mirror performance as a junior doctor.

To date predictive validity studies of the UKCAT have been few and produced conflicting results. With regards to the earlier years Lynch et al. [11] found no statistically significant associations between the Year 1 exam and UKCAT scores with 341 students from Dundee and Aberdeen medical schools. Yates and James [15] also examined potential predictive relationships in 204 pre-clinical Nottingham students but found very limited evidence in favour of the UKCAT, with the assessment explaining between 2 and 3 percent of the variance in exam scores. In contrast, Wright and Bradley [7] have presented positive results favouring the UKCAT, showing a modest predictive relationship with Year 1 and 2 knowledge exam scores among 304 Newcastle medical students. More recently, McManus et al. [16] also showed small predictive relationships between UKCAT scores and Year 1 OSCE and written exam scores in a large scale study of 4811 students from 12 UK medical schools.

To date only one published study has examined the predictive validity of the UKCAT in the clinical years. Yates and James [17] followed 185 students from the cohort reported on earlier [15] and found statistically significant correlations between .173 and .259 among both written and clinical examinations. However, when included in a hierarchical multiple regression with prior attainment in pre-clinical years as a predictor, the UKCAT was not found to be independently predictive of subsequent performance on the course. The authors concluded that the UKCAT remains ‘unproven, and requires wider investigation’ (p.1) [17]. However, there are concerns with this analysis. Most obviously, as prior attainment in pre-clinical years is not available at the point of selection, its use in assessing the efficacy of admissions tools is questionable. Furthermore, reliability data was not provided for the examinations under scrutiny. It is therefore clear that further research remains necessary to determine whether the UKCAT is a useful selection tool.

This study adds to the existing body of knowledge by examining the predictive validity of the UKCAT in the later years of medical school in relation to that of traditional pre-admissions selection tools. This is a follow-up study of students from two Scottish medical schools who were included in an earlier study examining the predictive validity of the UKCAT in Year 1 of their course [11].

## Methods

We followed up a group of students who entered into medical school at the Universities of Aberdeen and Dundee in 2007 from their first year to graduation in their fifth year.

Components of local admissions policies at both institutions were similar to those described by Parry et al. [18]. After being screened for minimum academic qualifications, UCAS form components were assigned numerical scores, namely research into medical careers, non-academic achievements as well as extra-curricular activities (all derived from the personal statement), academic qualifications and references. A total UCAS form score was calculated as a composite of UCAS form components. This composite score was used for selection into a traditional panel-style interview.

Offers were issued based on a combination of interview and UCAS form scores for Aberdeen and interview scores alone for Dundee. UKCAT scores were not used for admissions decisions by Aberdeen and were used at Dundee only to rank those near the cut-point for offers. For both institutions data collected at admissions included student demographics, UCAS form and UKCAT scores. Further details on the selection processes at the two schools are described in detail in our earlier paper [11].

The two institutions differ in the structure of their final year assessments. At both schools students sit written exams and objective structured clinical examinations (OSCEs) in year 4. However, in Dundee, students sit their final whole-cohort OSCE in year 4, followed by a portfolio assessment in year 5 which is based on a series of coursework submissions and results in a pass/fail decision. Aberdeen students have a further OSCE in year 5 and do not have a portfolio assessment. The portfolio submission was not used in this analysis as it is an inherently different assessment to written and OSCE examinations which are both comparable across institutions.

Both institutions supplied written examination and OSCE scores. Aberdeen’s written examination was an aggregate score of short answer questions (SAQs), extended matching questions (EMQs) and single best answer (SBA) questions. Dundee’s written examinations comprised of multiple choice questions. Raw scores from written examinations and OSCEs were recorded separately. In line with most similar studies [8,19] the authors prefer not to adjust for prior attainment as our research focus is on the association between of pre-admissions variables and medical school assessments.

As a measure of psychometric robustness Table 1 shows reliabilities for each assessment. All assessment reliabilities were calculated using Cronbach’s alpha with the exception of Aberdeen’s Year 4 Written assessments which was calculated using Kuder-Richardson 20. These figures broadly suggest acceptable levels of reliability for each assessment [20].

**Table 1:** Examination reliabilities

**Institution**	**Year 4 written**	**Year 4 OSCE**	**Year 5 OSCE**
Aberdeen	0.73 (EMQ), 0.74 (SBA), 0.76 (SAQ)	0.68	0.70
Dundee	0.92	0.72	N/A

Table 2 provides an overview of the study populations for both institutions. Routine data indicated that students with no UKCAT scores were exempt due to deferred entry or a prohibitive medical condition or geographic location preventing them from attending a UKCAT testing centre. Intercalated degrees are undertaken by students who opt to take a year out of their undergraduate medical course to study a subject area of their choice in greater depth, after which they return to complete their medical studies. These students effectively change cohort and hence could not be included in the study.

**Table 2:** Study populations for both institutions

	**Total matric**^**1**^	**No UKCAT**	**Intercalate**	**Withdraw/Repeat**	**Year 4**	**Year 5**
Aberdeen	174	10	31	44	99	89
Dundee	167	23	55	38	51	N/A

^1^Total number of students matriculated in Year 1.

Table 3 shows a breakdown of demographic data, namely gender and age group which was dichotomised prior to receiving the data into graduate/mature (G/M) candidates (aged 21 or older at the start of their studies) and school leavers (aged 20 or less) for reasons of data privacy. This distinction is relevant as age has been shown to be positively related to OSCE performance [21] and negatively associated with UKCAT score [22].

**Table 3:** Demographic data for both institutions

	**Aberdeen**				**Dundee**	
	**Y4**		**Y5**		**Y4**	
	N	%	N	%	N	%
Male	47	47.5	43	48.3	16	31.4
Female	52	52.5	56	62.9	35	68.6
Graduate/Mature	19	19.2	18	20.2	16	31.4
School leaver	80	80.8	71	79.8	35	68.6
*Total*	*99*	*-*	*89*	*-*	*51*	N/A

Of the 99 matched Aberdeen year 4 students, 47.5% were male and 52.5% female. 19.2% percent of this group were G/M and 80% were school leavers. Fewer students sat Aberdeen’s year 5 assessments where 43.3% were male and 62.9% were female. 20.2% of this group were G/M and 79.8% were school leavers. Of the 55 matched Dundee students 31.4% were male and 68.6% female. 31.4% of this group were G/M and 68.6% were school leavers.

### Analysis

Data were analysed with SPSS 19.0. Independent variables were admissions tools (total UKCAT, UCAS form and interview scores) and the demographic variable gender. Histograms and plots were used to confirm that the data were linear and normally distributed. Pearson’s correlations were used to test relationships between admissions tools and examination scores. Correlations were used to select variables for multiple linear regression analysis to predict examination scores. A significance level of p < 0.05 was required for a variable to be included in a multiple regression model.

Similar to other studies of this type which explore potential associations based on available data, we did not correct for family-wise error as they relate to multiple comparisons made [8,23,24]. The results presented should therefore be interpreted whilst bearing the possibility of Type 1 error in mind.

Forced entry multiple regression was performed, where all chosen predictors are forced into the model simultaneously. This procedure allows us to see the contribution of each independent variable to the model’s ability to predict assessment performance [25]. This procedure was data-driven as there was no theoretical basis for selecting variables used in the model. Where there was only one significant predictor for an assessment the result for the regression is equivalent to a simple linear regression.

Strength of correlations were compared using Cohen’s effect size interpretations [26] (small ≥ .10, medium ≥ .30, large ≥ .50) and the US Department of Labour, Employment Training and Administration’s guidelines for interpreting correlation coefficients in predictive validity studies [27] (‘unlikely to be useful’ < 0.11; ‘dependent on circumstances’, 0.11–0.20; ‘likely to be useful’ 0.21–0.35; ‘very beneficial’ > 0.35).

The University of Dundee Research Ethics Committee confirmed the study met the required University ethical standards but did not require ethical approval as it originated from an audit of admissions and selection procedures.

## Results

Tables 4 and 5 shows descriptive statistics as well as Pearson’s r correlations between admissions tools and examination scores for Aberdeen and Dundee respectively. Statistically significant correlations have been highlighted in bold.

**Table 4:** Descriptive statistics and correlations between pre-admissions measures and exam scores for Aberdeen

	**Measure**	**Mean**	**SD**	**1**	**2**	**3**	**4**	**5**	**6**	**7**
1	Gender^1^	0.47	0.50	-									
2	Age Group^2^	0.81	0.40	0.02	1.00								
3	UCAS score	23.80	3.10	0.07	0.06	1.00							
4	Interview Score	37.87	4.93	0.02	0.08	0.05	1.00						
5	UKCAT Total	2347.47	231.27	-0.01	0.09	-0.07	0.17*	1.00					
6	Y4 Written	71.28	5.57	-0.23*	0.04	-0.02	0.07	0.24*	1.00				
7	Y4 OSCE	74.07	5.02	-0.30**	0.15	0.00	0.10	0.36**	0.63**	1.00			
8	Y5 OSCE	81.05	4.47	-0.26*	0.02	0.14	0.02	0.29**	0.59**	0.68**			

*p < .05; **p < .01.

^1^Gender was coded as 1: Male, 0: Female.

^2^Age Group was coded as 1: Not Graduate/Mature, 0: Graduate/Mature.

**Table 5:** Descriptive statistics and correlations between pre-admissions measures and exam scores for Dundee

	**Measure**	**Mean**	**SD**	**1**	**2**	**3**	**4**	**5**	**6**
1	Gender^1^	0.29	0.46	1.00								
2	Age Group^2^	0.69	0.47	0.02	1.00							
3	UCAS score	13.14	0.60	-0.11	0.11	1.00						
4	Interview Score	56.86	2.64	-0.10	-0.08	0.01	1.00					
5	UKCAT Total	2437.65	208.59	-0.01	0.09	-0.05	-0.01	1.00				
6	Y4 Written	73.80	5.92	-0.04	0.06	-0.17	0.00	0.34*	1.00			
7	Y4 OSCE	72.26	5.53	0.04	0.07	0.14	0.07	0.15	0.59**			

*p < .05; **p < .01.

^1^Gender was coded as 1: Male, 0: Female.

^2^Age Group was coded as 1: Not Graduate/Mature, 0: Graduate/Mature.

Table 6 shows multiple regression statistics for each assessment for both institutions where there was a significant correlation with an admissions tool. While analysis revealed there was only one statistically significant relationship for Dundee’s year 4 written, model statistics for the multiple regression analysis was included in the table for consistency.

**Table 6:** Forced entry multiple regression statistics

**Institution**	**Assessment**	**Model statistics**				**Independent variables**			
		**R**^**2**^	**Adjusted R**^**2**^	**F**	**p**	**Predictor**	**B**	**Stand B**	**P**
Aberdeen	Year 4 Written	0.10	0.09	5.55	0.005	UKCAT	0.01	0.24	0.02
						Gender	-0.23	-0.21	0.03
	Year 4 OSCE	0.22	0.20	13.38	0.001	UKCAT	0.01	0.36	0.01
						Gender	-2.92	-0.29	0.01
	Year 5 OSCE	0.15	0.13	7.38	0.001	UKCAT	0.01	0.29	0.01
						Gender	-0.22	-0.25	0.02
Dundee	Year 4 Written	0.11	0.09	6.18	0.016	UKCAT	0.01	0.34	0.02

### Aberdeen

Significant positive correlations of .24 and .36 were seen between UKCAT score and the year 4 written and OSCE assessments respectively. There were no significant associations with either the interview or UCAS form scores. Females performed significantly better than males in both the year 4 OSCE and year 4 Written.

A significant positive correlation of .29 was observed between UKCAT score and the year 5 assessment. There were no significant associations with either the interview or UCAS form scores. Females significantly outperformed males.

Multiple regression analysis revealed statistically significant predictors. UKCAT score explained between 6 and 13 percent of the variance in each exam. The final regression models explained between 10-22% of the variance in each exam.

### Dundee

A significant positive correlation of .35 was observed between UKCAT score and the year 4 written assessment. There were no significant associations with any other admissions tools or gender.

Overall the UKCAT score explained 11% of the variance in year 4 exam scores.

## Discussion

This follow-up study examines the relationship between UKCAT scores and performance in the later years of medical school, specifically Years 4 and 5 of five-year programmes. We found supportive evidence for the assertion that the UKCAT is better at predicting performance in the later years than in the earlier years of medical school. This is in contrast to the pattern of decline observed in studies involving the MCAT [12,14] and with generally larger validity coefficients than reported by Yates and James [17].

While the correlations are small to medium they can be considered as ‘likely to be useful’ or ‘beneficial’ when interpreting the results of validity studies [27]. Furthermore, UKCAT score was the largest predictor of medical school academic performance in the multiple regression models, accounting for between 6 and 13 percent of the variance.

These findings have important implications given that the senior clinical years of medical school are more similar to those of a junior doctor than the early, preclinical years. The results from the Aberdeen OSCEs are particularly reassuring as the clinical situations representing these assessments are more similar to what is seen in clinical working practice. While the UKCAT was not predictive of Dundee OSCE performance, this may be because different teaching and assessment methods may lead to different results [7] and underscores the need for further multi-institutional studies.

We also identified that traditional interview and UCAS form scores appear to lack predictive validity, yielding no statistically significant positive associations. This is consistent with the body of evidence in this area which suggests that these admissions tools are not predictive of medical school outcome markers [6-8]. This adds to the general consensus worldwide that neither of these admissions tools add value to the selection process and calls their continued use into question [3,4,9].

This is a longitudinal follow-up study of two cohorts of students from two medical schools. It has some limitations. Both cohorts are small and the lack of statistical power could potentially reduce the generalizability of the results. The results should also be viewed with some caution because of the potential for Type 1 errors with multiple comparisons.

Despite these limitations the results of this study presents modest support for the use of the UKCAT in medical school selection. Future studies should examine the incremental validity of the UKCAT over academic criteria (such as A-levels/Scottish Highers) in predicting exam results, while taking into account makers of socioeconomic class. As cohorts mature, future research should also assess the utility of the UKCAT and other admissions measures into postgraduate study and workplace performance assessment. Evidence from other UK medical schools with more diverse curricula and summative assessments would be beneficial. Also while the UKCAT explains some of the variance, a substantial proportion remains unexplained. A multi-pronged approach to admissions measures should be investigated so the UKCAT is used in combination with other admissions measures such as the Multiple Mini-Interview (MMI) which have been shown to be predictive of medical school examination scores [8,19,28,29].

## Conclusions

Results suggest the UKCAT appears to predict performance better in the later years of medical school compared to earlier years and provides modest supportive evidence for the UKCAT’s role in student selection within these institutions. Further research is needed to assess the predictive validity of the UKCAT against professional and behavioural outcomes as the cohort commences working life.

## Abbreviations

GAMSAT: Graduate Australian Medical School Admissions Test; MCAT: Medical Colleges Aptitude Test; MMI: Multiple Mini-Interview; OSCE: Objective Structured Clinical Examination; UCAS: Universities and Colleges Admissions Service; UKCAT: United Kingdom Clinical Aptitude Test.

## Competing interests

JD is Dundee Medical School’s representative on the UK Clinical Aptitude Test (UKCAT) Board and therefore has an interest in demonstrating the value of the UKCAT.

## Authors’ contributions

All authors contributed to the study conception and design, the acquisition, analysis and interpretation of data, drafting and critical revision of the article. All authors read and approved the final manuscript.

## Pre-publication history

The pre-publication history for this paper can be accessed here:

http://www.biomedcentral.com/1472-6920/14/88/prepub
